# Opioids-Induced Long QT Syndrome: A Challenge to Cardiac Health

**DOI:** 10.1007/s12012-024-09853-6

**Published:** 2024-04-17

**Authors:** Jiale Hu, Yongfei Song, Xiaoyan Huang, Chongrong Li, Xiaojun Jin, Lichao Cen, Chuanjin Zhang, Beilei Ding, Jiangfang Lian

**Affiliations:** 1 Department of Cardiology, Ningbo University Health Science Center Affiliated Lihuili Hospital, Ningbo University, Zhejiang, China; 2 Ningbo Institute of Innovation for Combined Medicine and Engineering, Ningbo Medical Center Lihuili Hospital, Ningbo University, No. 378 Dongqing Road, Yinzhou District, Ningbo, 315000 Zhejiang China

**Keywords:** Opioids, Acquired long QT syndrome (aLQTS), QT interval, Ion channel

## Abstract

The challenge posed by opioid overdose has become a significant concern for health systems due to the complexities associated with drug prohibition, widespread clinical use, and potential abuse. In response, healthcare professionals have primarily concentrated on mitigating the hallucinogenic and respiratory depressant consequences of opioid overdose to minimize associated risks. However, it is crucial to acknowledge that most opioids possess the capacity to prolong the QT interval, particularly in cases of overdose, thereby potentially resulting in severe ventricular arrhythmias and even sudden death if timely intervention is not implemented. Consequently, alongside addressing the typical adverse effects of opioids, it is imperative to consider their cardiotoxicity. To enhance comprehension of the correlation between opioids and arrhythmias, identify potential targets for prompt intervention, and mitigate the hazards associated with clinical utilization, an exploration of the interaction between drugs and ion channels, as well as their underlying mechanisms, becomes indispensable. This review primarily concentrates on elucidating the impact of opioid drugs on diverse ion channels, investigating recent advancements in this domain, and attaining a deeper understanding of the mechanisms underlying the prolongation of the QT interval by opioid drugs, along with potential interventions.

## Introduction

Long QT syndrome (LQTS) is a heterogeneous group of disorders caused by cardiac repolarization maladaptation [1]. This dysfunction is characterized by a prolonged QT interval on electrocardiographic (ECG) readings and is associated with an increased risk of adverse cardiac events, such as tip-twisting ventricular tachycardia (TdP), recurrent syncope, cardiac arrest, and potentially sudden death [1]. LQTS can be classified as hereditary or acquired. Acquired LQTS (aLQTS) occur in approximately 0.7% of hospitalized patients and are often linked to underlying medical conditions or the use of certain medications [2].

The pathogenic of aLQTS are mainly external factors. Common causes include electrolyte disturbances such as hypokalemia, congestive heart failure, coronary heart disease, diabetes mellitus, myocardial ischemia, and some QT-prolonging medications [2]. These factors often lead to functional alterations in various potassium and calcium channels [3–6], such as Kv11.1 potassium selective channels encoded by human cardiac Ether-à-go-go–related gene (hERG), potassium voltage-gated channel subfamily Q member 1, ATP-sensitive potassium channels, Kir3.1 potassium channels, transient outward potassium channels and Long-lasting calcium channel (L-CaV). Among these causes, drugs play a significant role in triggering aLQTS [7]. Known drugs that can cause LQTS include antiarrhythmics, antineoplastics, antidepressants, nonsteroidal analgesics, and opioid analgesics, among others [8]. However, most current studies only focus on the phenotypic level, leaving the mechanisms of drug-induced action potential alterations not fully understood. Additionally, the response to the same drug can vary among individuals, possibly due to the interaction between drug-specific and patient-specific risk factors, or differences in individual repolarization reserve, which is influenced by their genetic background. Consequently, the identification of potential loci for the risk of serious arrhythmia in susceptible individuals is a major focus of current research [9].

Opioid analgesics have been extensively utilized for managing acute severe pain and cancer pain ever since they were first derived from opium poppy (*Papaver somniferum*) for postoperative analgesia in 1784 [10]. Commonly used opioid drugs include synthetic opioid drugs like buprenorphine, methadone, morphine. Synthetic opioid drugs are less addictive than natural opioid drugs, but they still have many of the same side effects. According to the 2023 World Drug Report released by the United Nations Office on Drugs and Crime, the number of people using drugs has increased by 23% from 2011 to 2021. Opioids have one of the highest rates of all serious drug-related injuries [11]. In addition to the widespread clinical use of opioids, repeated discontinuation also leads to a higher incidence of opioids side effects. The common toxic effects of opioid drugs are strong addiction and ventilatory inhibition [12]. This explains why medical staff focus more on avoiding drug overdose and preventing ventilatory depression but ignore the possibility that opioids may cause cardiovascular disease (CVD) in clinical practice. One of recent retrospective studies shows that opioids users have significantly higher risk of hospitalization, ischemic stroke and death from heart failure compared with non-users [13]. Among these, TdP, a polymorphic ventricular tachycardia with mandatory QT-interval prolongation, accounts for a large proportion of cardiovascular deaths.

Evidence is mounting that opioids may trigger cardiovascular events and life-threatening arrhythmias like tip torsion ventricular tachycardia, but knowledge of opioid-induced QT-interval prolongation is still largely limited to its effects on ion channel function, with less involvement in the transcriptional expression of ion channels. Here, the arrhythmogenic effects and risk factors of opioids, the role of opioid receptor-mediated ion channel dysfunction, and possible countermeasures will be briefly described.

## Opioids-Induced QT-Interval Prolongation

It has been reported that opioids can cause QT-interval prolongation, which in turn develops into TdP and even sudden death [6, 14–19]. Opioids are categorized into three types. Natural opioids refer to the natural alkaloids and active ingredients isolated and analytically purified from*Papaver somniferum*, commonly including morphine and codeine. Semi-synthetic opioids are derivatives derived from the synthesis of natural opioid alkaloids, such as heroin. Drugs that are synthesized directly in the laboratory are known as synthetic opioids, such as methadone and pethidine [20]. Different types of opioids have different addictive properties and safety profiles. The natural drug morphine and the semi-synthetic drug buprenorphine are less likely to prolong the QT interval, while the synthetic opioids are more likely to cause alterations in myocardial QT [6]. The impact of opioids on cardiac function is complex and heavily influenced by patient exposure. Prolonged opioids use, abuse, overdose, and withdrawal all contribute to the development of complications. loperamide can lead to cardiac arrest or sudden recurrent syncope with ECG abnormalities, including significant prolongation of the QT interval, widening of the QRS interval, and ventricular arrhythmias, as well as conventional opioid toxicity [19]. Increased nocturnal mortality among methadone users may be attributed to cardiac repolarization instability resulting from prolonged use and sleep-related hypoxemia [18]. A retrospective study in Iran confirmed that although methadone is much less addictive than opium, both are similarly likely to cause aLQTS [14]. This suggests that the effect of opioid drugs on aLQTS may not be particularly associated with their addiction. Moreover, in a cohort study of 137 patients with oxycodone overdose, 20 patients had an abnormal QT interval and 24 patients had bradycardia, including 5 with HR < 50 beats/min [21]. Compared with synthetic opioids such as methadone, which significantly affect QT interval, morphine is safer. Although some reports of morphine causing TdP have been reported, there is no evidence to confirm that morphine has an effect on QT interval [22, 23]. Although there is much clinical evidence to support the idea that opioids cause aLQTS, the exact molecular mechanism of pathogenesis remains to be clarified.

## The Risk Factors of Opioids Mediated aLQTS

### Combined Pharmacotherapy

Some reports indicate that the arrhythmogenic effects of opioids are amplified when they are used in the presence of some underlying disease, or when they are taken concurrently with some drugs. A cross-sectional study of 6790 hospitalized patients confirmed a significant increase in the propensity for aLQTS and other arrhythmias when patients with hypokalemia, abnormal T-wave morphology, hepatitis C virus infection, and human immunodeficiency virus (HIV) infection were taking specific psychotropic drugs and methadone [24]. In a prospective, open-label study of 50 HIV-negative, opioid-dependent subjects, no significant prolongation of the QT interval was observed in opioid-dependent individuals taking buprenorphine/naloxone alone, whereas the QT interval was increased with the combination of delavirdine or ritonavir [25]. In addition, cocaine, a substance frequently abused by opioid-dependent patients, may synergize with methadone to trigger TdP [26]. In the list of medications for geriatric outpatients, psychotropic drugs often cause harmful polypharmacy interactions that lead to 5-hydroxytryptamine syndrome, convulsive seizures, prolonged QT interval, and hemorrhage [27]. This suggests that it is important to assess the patient’s condition and past medical and medication history before applying opioids. Strict control of indications, cautious use of drugs in some patients with high-risk underlying diseases, and avoidance of drugs with harmful drug interactions will help reduce the risk of arrhythmias with opioids.

### Genetic Mutations

Genetic and metabolic differences are likewise among the major factors influencing aLQTS. Mutations in genes encoding ion channels resulting in malfunction of ion channels are among the most common causes. Multiple KCNH 2 mutations can significantly affect the sensitivity of hERG to drug blockade, leading to a prolonged QT interval [28–30]. The risk of QTc prolongation with methadone application is significantly increased in people with CYP2B6 hypometabolism, the first reported genetic factor affecting methadone metabolism and a locus associated with the risk of potentially serious arrhythmias and sudden death [31]. Therefore, genetic screening is important not only in the diagnosis of hereditary long QT syndrome, but also in the identification and management of aLQTS.

### Molecular Conformation

Ansermot et al. found a lower risk of cardiotoxic effects and sudden death with (R)-methadone by following 39 patients on long-term (R,S)-methadone and (R)-methadone [32]. This interesting result suggests that homologous drugs with different molecular conformations may have inconsistent cardiotoxicity. Relevant studies and papers are less reported, leading to the possibility that this result may not be universal. But finding the molecular conformation with the highest safety profile might be a novel measure to prevent drug side effects.

## Activation of Opioid Receptors Affects aLQTS

Opioid receptors mainly include μ, κ and δ receptors and opioids mainly regulate the function of the cardiovascular system by affecting them (Table1) [33]. Although opioid receptors are divided into multiple subtypes, they are structurally similar and have overlapping physiological effects [34]. These opioid receptors belong to transmembrane G protein coupled receptors (GPCR), whose signal transduction kinases cascade activates to achieve various protein changes [35]. The main effects of opioid receptors are known to be analgesia, euphoria, constipation, and respiratory inhibition, which are used for many clinical purposes, including analgesics, antidiarrheics, and cough drugs. In neuronal cells, all three opioid receptors inhibit pre- and postsynaptic Ca^2+^channels, thereby attenuating neuronal excitability and reducing the release of proinjurious neuropeptides [36]. In addition, opioid receptors activate G protein-coupled inwardly rectifying potassium (GIRK) channels, which contribute to neuronal excitation and action potential propagation [37]. But this effect has not been found in cardiomyocytes.

**Table 1:** Opioid receptors and effects

Receptors	Site of action	Effects
μ	Systemic	Analgesia, euphoria, constipation, respiratory depression
	Cardiovascular	Heart rate ↓, blood pressure ↓
κ	Systemic	Analgesia, diuresis, dysphoria
	Cardiovascular	Ischemic pre-conditioning
δ	Systemic	Analgesia, convulsions, anxiolysis
	Cardiovascular	Ischemic pre-conditioning

Opioid receptors also affect many systems. For example, opioid receptors can regulate cell growth, inflammation, and wound healing [38, 39]. Similarly, opioid receptors have been shown to have effects on the cardiovascular system, leading the decrease of heart rate and blood pressure and ischemic pre-conditioning [40, 41]. Cardiac μ-opioid receptors are significantly upregulated during heart failure [40]. Although there is currently insufficient evidence to prove that opioid receptors regulate ion channels in cardiomyocytes, based on the effect of different receptors of opioids on heart rate and their modulation of ion channels in neuronal cells, we can hypothesize that opioid receptors might contribute to aLQTS.

In addition to the three receptors mentioned above, there is also an opioid receptor called nociceptin/orphanin FQ peptide (NOP) receptor. Endogenous opioid substances such as endorphins exist in heart tissue, which provide some evidence for opioid receptors to mediate cardiac function [42]. Endogenous opioid systems have the ability to resist arrhythmia. Research has confirmed that the upregulation of endogenous opioid peptide secretion caused by short-term cholestasis is associated with the resistance to ischemia/reperfusion-induced arrhythmia [43, 44]. However, there are few relevant studies, and the specific mechanism of how endogenous opioid peptides affect the heart through the expression of opioid receptors remains unclear and needs to be explored.

## Opioids and Ion Channel

The essence of arrhythmia is the alteration of the action potential (AP) of cardiomyocyte. AP in working myocytes can be divided into 5 distinct phases, including the rapid depolarization (0), transient repolarization (1), plateau (2), rapid repolarization (3), resting potential or final repolarization (4) [45]. Abnormalities in the function of ion channels due to any cause can lead to abnormalities in the AP of cardiomyocyte, that can manifest as visible alterations on the ECG.

In patients with hereditary LQTS, the prolongation of the QT interval may be due to a prolongation of the basal action potential duration (APD) due to genetic mutation-induced ion channel dysfunction resulting in decreased activity of repolarizing slowly activating delayed rectifier potassium current (*I*_Ks_) or delayed rectifier potassium current (*I*_Kr_) or sustained sodium influx into the plateau phase [46]. The mechanism of aLQTS is similar, i.e., drugs ultimately affect the function of ion channels through various pathways. The current research focuses on hERG channels, which affect the QT interval most significantly. Recent studies have also identified new mechanisms and ion channel loci, I_K1_for example [16, 17, 47].

### Potassium Currents

Kv11.1 potassium selective channels encoded by hERG is the most studied aLQTS ion channel, which mediates*I*_Kr_[48].*I*_Kr_plays an important role in cardiac action potential repolarization. The inhibition of*I*_Kr_function predisposes ventricular myocytes to early afterdepolarizations (EADs) and may induce delayed afterdepolarizations (DADs), which may be the basis for subsequent malignant arrhythmias. Genotyping of hERG is associated with prolonged QT interval in patients on methadone maintenance therapy. Among them, each Lys allele copy at codon 897 of KCNH2 was most closely associated with QT-interval prolongation [30]. In addition to methadone and LAAM, both fentanyl and orlistat have now been validated in cytologic experiments for possible inhibition of hERG channels, prolongation of the QTc interval, and increased susceptibility to sudden cardiac death [49].

**Fig. 1:**
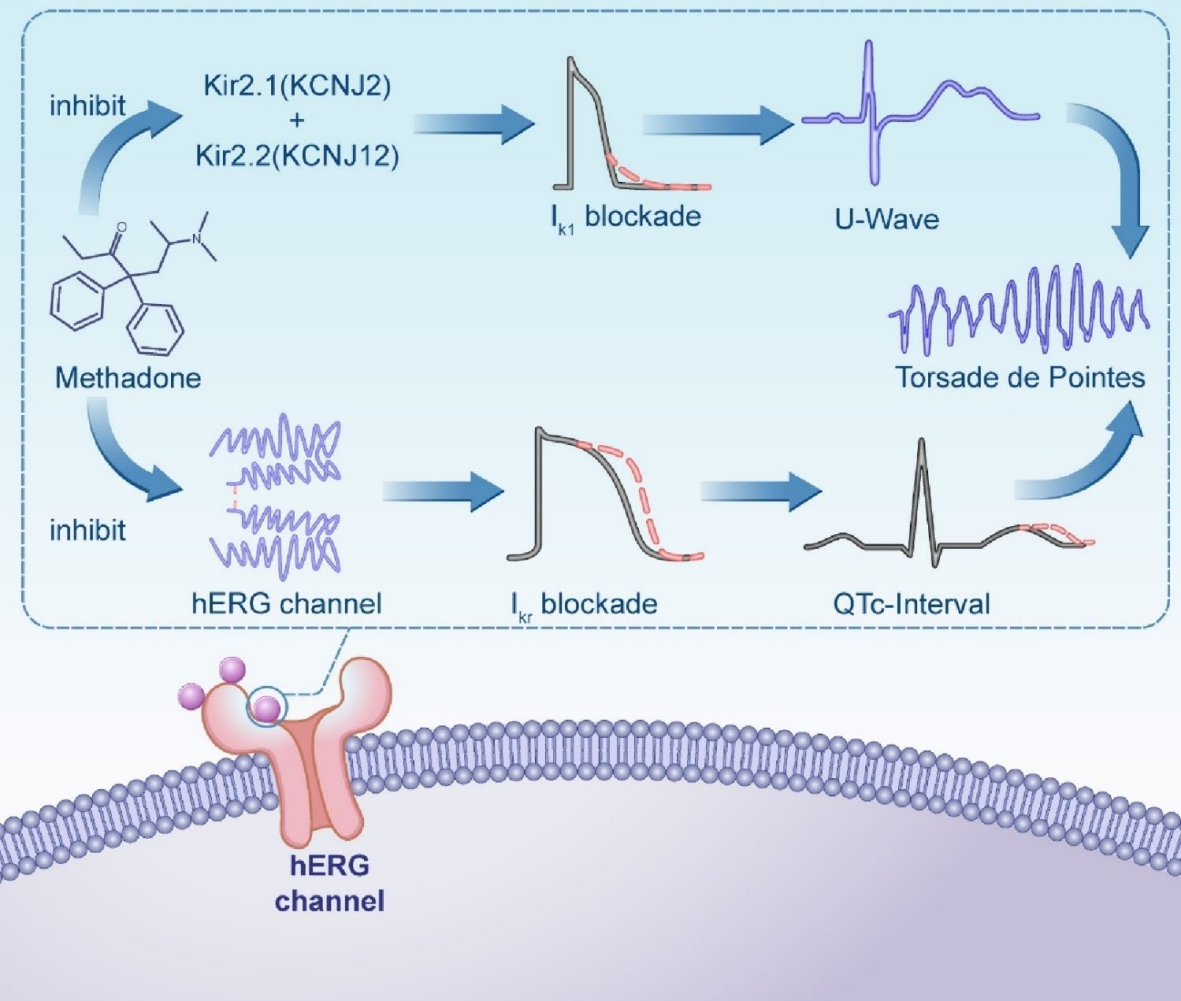
A schematic diagram illustrates the molecular mechanisms through which methadone induces arrythmias

Many drugs have been shown to inhibit hERG channel function. The mechanisms behind this are worth exploring. Methadone and its derivative levacetalymethadol (LAAM) are synthetic opioids containing two hydrophobic aromatic rings attached to a conformationally flexible molecular framework capable of blocking hERG (Fig.1) [50]. The inhibition of*I*_Kr_due to blocking hERG channels results in prolonged cardiac repolarization. This is reflected on an electrocardiogram as a prolonged QT interval and can lead to life-threatening ventricular tachyarrhythmia, specifically tip torsion ventricular tachycardia [51]. In contrast, semi-synthetic and natural opioid drugs lack this double aromatic ring structure but still cause QT interval prolongation [33], suggesting that other mechanisms may be involved.

One possibility is opioid receptors. Regrettably, because of the lack of attention to the arrhythmogenic effects of opioid drugs, most studies of the effects of opioid receptors on ion channels have focused on ion channels expressed on the membranes of neuronal cells and respiratory smooth muscle cells. The specific role of opioid receptors in drug effects on potassium channels, particularly hERG channel, is currently a major challenge in the field. Constitutive interactions between DORs, Gβγ and Kir3 subunits have been identified, but it is not yet clear whether there are further effects on channel function [52]. Although the evidence is limited, the role of opioid receptors in aLQTS cannot be ignored.

The main role of the inward rectifier*K*^+^current (*I*_K1_) is to regulate intracellular potassium concentration, maintain intracellular and extracellular electrochemical balance. The inhibition of I_K1_, a major determinant of the final repolarization phase, results in prolonged repolarization and shortened post-repolarization refractory period, but has no significant effect on the total duration of AP [53]. A translational study conducted in 2022 demonstrated that methadone, a potent inhibitor of*I*_K1_, can lead to an increase in U waves on surface ECGs [16] (Fig.1).

Methadone’s bi-aromatic ring structure binds to the internal site of the hERG channel, blocking*I*_Kr_, delaying repolarization and prolonging action potential duration, which means a prolongation of the QT interval, while methadone induces*I*_K_-channel block resulting in changes in the U-wave [50, 53]. The alteration of the QT interval and the U-wave may lead to a variety of ventricular arrhythmias, which may ultimately progress to TdP, causing the patient’s sudden death.

### Sodium Currents

The voltage-gated sodium channels (NaV) channel is responsible for action potential amplitude, conduction velocity, and propagation [54]. NaV1.5 can initiate depolarization of AP. Activation of NaV1.5 leads to an increase in inward sodium current and results in prolonged QT intervals and APD [55]. At the same time, in the presence of prolonged APD, cardiomyocytes tend to develop EADs, which can trigger TdP [56].

Oxycodone has been found to cause QT-interval prolongation of the in clinical practice, but it induces weak hERG channel blockade. Whole-cell patch-clamp experiment confirmed that oxycodone leads to an accumulation of NaV1.5 channels in inactivated states in a significant concentration dependent manner. Furthermore, human stem cell-derived cardiomyocytes had abnormally low heart rates and severe arrhythmias under high-concentration drug interventions [47]. Methadone is also present to inhibit the function of NaV1.5 channels through interaction with LA-binding [47]. Similarly, tramadol reduces NaV1.5 currents, in particular, at close-to-physiological membrane potentials [57]. However, no functional effect of buprenorphine on NaV1.5 channels could be detected at clinically relevant concentrations [15].

Interestingly, most opioid drugs activate NaV1.5 function, which does not directly lead to prolongation of the QT-interval. Experiments with multiple concentration gradients revealed that the activity of opioid drugs acting alone on hERG or Nav1.5 channels at pharmacological concentrations may not be sufficient to cause cardiac side effects in patients [17]. However, their combined action significantly amplifies the effects on the excitability of cardiomyocytes. Blockade of hERG channels leads to prolonged depolarisation, inducing slow inactivation of Nav1.5 channels. This further increases the number of inactivated Nav1.5 channels, leading to disturbed myocardial rhythms and QT-interval prolongation.

In summary, one opioid drug can affect multiple ion channels, and this effect is complex and requires comprehensive and careful analysis.

### Calcium Currents

L-CaV and transient calcium channel (T-CaV) are the two main calcium channels in the myocardial cell membrane. L-CaV is the main pathway of calcium inward flow during cell excitation and is closely related to excitation-contraction coupling and excitation-secretion coupling. T-CaV participates in the pacing activity and repetitive delivery of myocardial sinus node and neurons, maintains cellular autoregulation, and is associated with transmembrane movement of calcium from calcium channels during low membrane potential [58]. In hereditary LQTS, type 4 and type 8 are associated with calcium channel. LQTS type 8 is an L-CaV disease with intracellular calcium overload and prolonged action potential time course due to mutations in CACNA1C, the gene encoding the L-CaV1.2 [59].

Opioids that have now been shown to affect L-CaV function include loperamide and methadone. They can interfere with hERG channel, NAV1.5 and others at the same time [15, 60]. This explains the high arrhythmogenic risk of methadone and loperamide among all opioids. Although research is scarce and only a few opioids have been shown to inhibit CaV, calcium currents cannot be ignored as an important component of AP.

## Low-Addictive Opioids also Carry Arrhythmia Risk

Evidence shows that low-addictive opioids have less side effects on the nervous and respiratory system of users, but the risk of arrhythmia is similar to methadone, a representative of synthetic opioids, which are currently recognized to have the greatest impact on heart rhythm [33]. At the same time, low-addictive opioids are more easily accessible to patients, with a relatively high risk of abuse and misuse. Therefore, the arrhythmogenic risk of low-addictive opioids cannot be ignored.

### Dextromethorphan

Dextromethorphan, the right-handed isomer of the morphine analog levorphanol methyl ether, is a potent central cough suppressant but has no analgesic effect. Recent studies have surfaced that dextromethorphan, in addition to its cough suppressant effects, may have a favorable therapeutic effect on major depression in adults [61, 62]. In the past, the drug was considered to have a low addictive potential and mild side effects and was once marketed and applied as an over-the-counter drug. However, a recent clinical case suggests a possible induction of prolonged QT interval with dextromethorphan: a 27-year-old white male with no significant past medical history developed syncope, hypokalemia, and prolonged QT interval after ingestion of approximately 27 mg/kg dextromethorphan and ethanol, and the prolonged QT interval was confirmed to be unrelated to alcohol ingestion at follow-up as well as in previous follow-ups [63]. To date, relatively few clinical reports of dextromethorphan-induced QT-interval prolongation have been published, and there is a gap in mechanistic studies, which needs to be filled as a commonly used drug in clinical practice.

### Tramadol

Tramadol is a centrally acting weak μ-opioid receptor analgesic, mostly used for the relief of moderate to severe pain, with much less addictive properties and incidence of adverse effects than morphine, and is a commonly used class of drugs in clinical practice [64]. Hydroxychloroquine has seen a significant increase in use since the COVID-19 pandemic compared to its previous use. It has an inherent side effect of prolonging the QT interval, however, when hydroxychloroquine is combined with tramadol, the risk of QT-interval prolongation is exacerbated [65]. However, for tramadol alone, a mild overdose application of tramadol hydrochloride did not cause an increase in the QTc interval in healthy adults as shown in a randomized, double-blind, multi-dose study [66].

### Loperamide

Loperamide is an over-the-counter opioid, similar in structure to haloperidol and pethidine, which inhibits the contraction of intestinal smooth muscle and reduces intestinal peristalsis, and is mainly used for acute and chronic diarrhea of various etiologies, especially for the long-term treatment of chronic diarrhea [67]. As mentioned earlier, loperamide affects multiple ion channels when applied at high doses, causing ECG changes such as prolonged QT interval [60]. Due to the large number of loperamide users and the increasing number of cases of loperamide poisoning, loperamide poisoning can now be treated with naloxone. However, naloxone may not effectively suppress loperamide-induced QT interval , and it has even been reported to further prolong the QT interval in patients [68]. Therefore, the current priority is to strictly control the use of loperamide and avoid drug overdose as much as possible. In addition, the need for regular monitoring of the ECG in patients applying the drug has yet to be agreed upon.

## Conclusion

The arrhythmic complications of opioids are a public health problem that cannot be ignored. Affecting multiple ion channel functions is the main mechanism by which most opioids cause QT-interval prolongation, with hERG channels being the main target of their action [33]. In addition, factors such as patient history, genotype, and conformation of the drug have varying degrees of influence on the arrhythmogenic effects of opioid drugs. To reduce the risks involved, physicians should carefully control the use of medications, with strict indications and prescription amounts, and avoid long-term prescriptions whenever possible. Patients' pre-existing medical conditions and medication histories should be kept in mind to prevent serious consequences from disease progression or drug interactions. In addition, timely identification of dependence and withdrawal is essential to ensure early treatment. There is controversy as to whether ECG monitoring must be performed in patients on opioids, but the collaborative guidelines of the American Heart Rhythm Society and the American Pain Society recommend screening for ECG in people with chronic pain on methadone to reduce the risk of drug use [69].

In a further way, studies on the molecular mechanisms of opioid-induced QT-interval prolongation are limited and superficial, which is very detrimental to our better understanding of the aLQTS. The application of induced pluripotent stem cells (iPSC) technology directed differentiation of myocardium with similar biological properties and functions as cardiomyocytes in vivo is a credible in vitro model for the study of human cardiac diseases. In recent years, it has been widely used in many aspects of LQTS pathogenesis research, drug screening, tissue engineering, etc [70–72]. It is also an effective tool that can be used for future research on the molecular mechanism of opioid drugs.

In conclusion, besides delving into the mechanisms underlying drug pathogenesis and seeking possible therapeutic targets, the most important strategies for reducing opioid cardiac risk are careful prescription formulation, timely detection of unexpected cardiovascular events, and arrhythmia risk stratification of susceptible patients on long-term opioid therapy.

## Data Availability

Not applicable.
